# Photocrosslinkable Morin-loaded gelatin-g-GMA composite hydrogel for accelerating burn wound healing: *in vitro* and *in vivo* assessments

**DOI:** 10.1039/d5ra09621a

**Published:** 2026-01-26

**Authors:** Amr Negm, Samar A. Salim, Tasneem Abed, Marwa Mosaad Shakweer, Esraa B. Abdelazim, Jong Yeog Son, Yasser A. Elnakady, Mahmoud Elsabahy, Elbadawy A. Kamoun

**Affiliations:** a Department of Chemistry, College of Science, King Faisal University Al-Ahsa 31982 Saudi Arabia anegm@kfu.edu.sa ekamoun@kfu.edu.sa badawykamoun@yahoo.com; b Nanotechnology Research Center (NTRC), The British University in Egypt (BUE) El-Sherouk City Cairo 11837 Egypt; c Badr University in Cairo Research Center, Badr University in Cairo Badr City Cairo 11829 Egypt mahmoud.elsabahy@buc.edu.eg; d Department of Pathology, Faculty of Medicine, Badr University in Cairo 11829 Cairo Egypt; e Department of Pathology, Faculty of Medicine, Ain Shams University Cairo Egypt; f Department of Applied Physics and Institute of Natural Sciences, College of Applied Science, Kyung Hee University Suwon 446-701 Republic of Korea; g Department of Zoology, College of Science, King Saud University B.O. Box 2455 Riyadh 11415 Saudi Arabia

## Abstract

The impaired skin regeneration, scarring, and delayed healing make the management of burn injuries a challenging task. We designed a photopolymerized hydrogel of gelatin-grafted with glycidyl methacrylate (GMA) for burn management applications. Hydrogel was incorporated with Morin, a plant flavonoid that was originally isolated from the *Moraceae* family, with known anti-antioxidant and anti-fibrotic activities. The physicochemical characterization of the resultant hydrogel, including its gelation time and swelling properties, was conducted. The characterization results indicated that the hydrogel development was successful, exhibiting well-established porosity, as evidenced by the SEM images. *In vivo* evaluation demonstrated improved tissue regeneration characterized by enhanced collagen deposition and dermal re-modelling. Additionally, histopathological analysis indicated reduced fibrotic features and accelerated wound closure. Moreover, the hydrogel promoted epithelial regeneration, accelerating the closure of burns in a burn rat model. Furthermore, *in vitro* studies using a THP-1-derived M1 macrophage model, showed that the Morin-loaded hydrogel formulations GH-5, GH-6, and GH-7 demonstrated a potent, concentration-dependent suppression of key M1 inflammatory mediators including nitric oxide (NO), IL-1β, and IL-6. This anti-inflammatory effect was mechanistically linked to the downregulation of critical genes (iNOS, COX-2, and STAT-3) that drive the M1 phenotype. Notably, the hydrogel with the highest Morin concentration (GH-7, 5%) exhibited the most significant reduction in inflammatory outputs, suggesting that the therapeutic efficacy is enhanced by Morin loading onto nanofibers. Collectively, this study provides a foundation for the development of functional hydrogels in regenerative medicine and tissue engineering, particularly in relation to burn therapy and modulating macrophage-driven inflammatory pathologies.

## Abstract 2

Gelatin–GMA hydrogels were synthesized by photopolymerization, characterized morphologically, and evaluated *in vitro* and *in vivo*, demonstrating immunomodulation, macrophage regulation, and enhanced wound healing.

## Graphical abstract

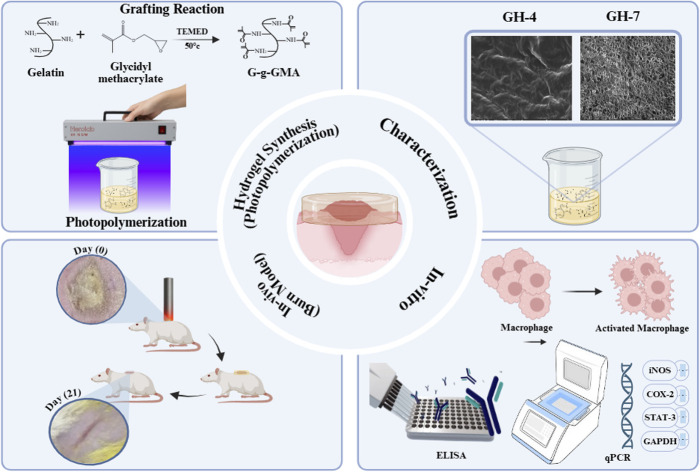

## 1. Introduction

The skin, the largest organ in the human body, has a surface area of approximately 1.5 to 2 m^2^ and weighs approximately one-seventh of the body's total weight. It is composed of 3 layers: the epidermis, dermis, and subcutaneous tissue.^1^ One of its most critical functions is as a barrier against invasion by external sources, such as viral, fungal, or microbial infection caused by pathogenic or opportunistic bacteria that reside on the skin.^2^ Compromises of this barrier, *via* wound or burn injury, compromise the body's defenses and can lead to infection, inflammation, or sepsis. Burn injury, a global phenomenon, accounts for one of the most common etiologies of the barrier breach. Burn injury, according to the WHO, causes 180 000 deaths annually, with thermal burns accounting for 70% of all burns, and being particularly frequent in children and the elderly.^3,4^ Burns are classified into four degrees: 1st-degree burns, which only injures the epidermis, is frequently red, painful, and dry with no blisters; 2nd-degree burns, which injure deeper into the epidermis, causes blisters, is painful, swollen, and represents 85.4% of burns; third-degree burns, which destroy both the epidermis and dermis and require surgery; and fourth-degree burns, the most severe, which pass through all skin layers and muscles and bones and can lead to organ loss or death.^5–7^ The study employs second-degree burns *via* thermal injury as the model to determine therapeutic efficacy. Traditional treatments utilize gauze dressing for burn wounds. The potential drawback of the practice, however, is that gauze adheres to the wound, and secondary trauma and pain upon dressing changes occur.^8^ This limitation has prompted the search for alternative means of improving burn care.

Hydrogels are a noninvasive and more efficient alternative to traditional wound dressings like gauze, with better biocompatibility, water retention, and drug release control. Unlike traditional dressings that adhere to the wound and cause trauma when being removed, hydrogels create an aqueous interface that allows tissue regeneration under conditions of less discomfort and mechanical trauma.^9^ Their tunable, porous nature enables the sustained and localized delivery of therapeutic agents, promotes wound healing and tissue regeneration, and prevents infection. They may also be engineered to be responsive to biological stimuli to achieve controlled drug release.^10^ Hydrogels may be either physically or chemically crosslinked, with the latter possessing enhanced mechanical stability.^11,12^ Among these, photo-crosslinked hydrogels have been of interest as they would be able to form stable, biocompatible constructs under mild polymerization conditions.^13,14^ Photopolymerized hydrogels, particularly those initiated by UV light, are a promising platform for drug delivery and burn wound healing. They can be gelled in place rapidly with mechanically tunable properties, allowing precise spatial control without the need for toxic chemical initiators, and thereby minimizing cytotoxicity.^14,15^ Their conformation to nonplanar burn wound beds, in combination with controlled release of therapeutic agents such as antimicrobial medications, growth factors, and anti-inflammatory drugs, enhances wound healing and minimizes systemic side effects. Photopolymerized hydrogels can also be environmentally sensitive to pH or temperature, thereby optimizing drug delivery and mechanical properties for mechanical integrity and controlled degradation.^16^ These multi-functional qualities make photopolymerized gelatin-GMA hydrogels attractive for future burn wound dressings and regenerative medicine.

Gelatin, a natural origin collagen, has versatile uses in biomedical applications due to its excellent biocompatibility, biodegradability, and bioactivity. However, it possesses poor mechanical strength and exhibits rapid degradation, which limits its utility for extended applications.^17^ To overcome these limitations, chemical modifications such as grafting with glycidyl methacrylate (GMA) have been studied. GMA grafts methacrylate groups onto the gelatin backbone such that it crosslinks when it is exposed to UV light. This modification improves the structural rigidity of the hydrogel, which is further suitable for application in wound healing.^18,19^ The gelatin-GMA hydrogel system provides a framework that is amenable to cell adhesion and growth and still has the needed mechanical and swelling properties for effective wound covering.^19^

Morin, a flavonoid present in most fruits and medicinal herbs naturally, has potent anti-inflammatory, antioxidant, and antimicrobial properties and is therefore very promising as a bioactive compound for burn treatment^20^ Burn injury tends to induce excess oxidative stress and inflammation, which can suppress healing and enhance the risk of infection. Morin is counteractive to these by neutralizing free radicals, inhibiting pro-inflammatory cytokines, and inducing fibroblast proliferation to improve tissue regeneration.^21^

Recent studies have highlighted the potential of gelatin-based hydrogels for loading bioflavonoids in various biomedical applications.^22–24^ Che zain *et al.* fabricated several polysaccharide/gelatin amorphous hydrogels impregnated with oil palm leaf-derived total flavonoid-enriched extract (OPL-TFEE) using a one-pot synthesis method. These hydrogels exhibited multiple crosslinking networks, demonstrating significant antioxidants and wound healing properties without any signs of cytotoxicity.^25^ In another study, flavonoids extracted from hawthorn leaves were loaded into gelatin hydrogels that were crosslinked with the natural biological crosslinker genipin. This resulted in a gelatin-genipin hydrogel carrier designed for the slow release of nerve growth factor and total flavonoids at spinal cord injury sites.^26^ Despite the recognized importance of flavonoids in medical applications, there remains a gap in research focusing on photopolymerized hydrogels for the treatment of burn injuries. Therefore, this study aims to incorporate Morin into a hydrogel matrix to enhance the healing process of burn wounds. The goal is to modulate the inflammatory response and promote tissue repair while providing an antimicrobial barrier against common pathogens associated with burn wounds. This innovative system combines the benefits of hydrogel-based wound dressings with the bioactive properties of Morin, representing a promising approach for burn treatment.

## 2. Materials and methods

### 2.1 Materials

Gelatin Type A, glycidyl methacrylate (GMA), and DMSO were obtained from Sigma Aldrich (St. Louis, MO), Irgacure 2959 (2-Hydroxy-4′-(2-hydroxyethoxy)-2-methylpropiophenone) (I_2959_) was obtained from (Merk, Germany), Morin (Combi blocks, USA) and acetone were purchased from (Qualikems, India), UV-lamp (16 watt, *λ* 365 nm, 0.08 Amps) (Model-Upland, CA, USA).

### 2.2. Methods

#### 2.2.1. Grafting reaction of G-g-GMA

The following procedure was followed to prepare gelatin grafted with GMA according to the previous method, with some modifications as reported elsewhere.^19^ The molar ratio was used to prepare a ten percent gelatin solution, which was then dissolved in 10 mL DMSO under continuous stirring and heated at 50 °C until the gelatin was fully dissolved, and a viscous/clear solution was formed. A definite amount of GMA (0.426 mL) was added to the gelatin solution to establish a molar ratio of gelatin to GMA (G/GMA) of (∼1 : 0.3 M). Tetramethylethylenediamine (TEMED) was added to the grafting solution as a catalyst in 1.0 mol% to enhance the grafting reaction between gelatin and GMA. The grafting reaction proceeded for four hours at low stirring and 50 °C, after which the addition of acetone terminated it, and G-g-GMA was precipitated as a sticky, white precipitate. Further washing with acetone was used to remove any excess amount of GMA.

#### 2.2.2. Optimization of G-g-GMA hydrogel

G-g-GMA hydrogel was prepared as previously reported by^15^ with some modifications. Briefly, (20% w/v) of the graft macro-monomer was redissolved in a mixture of distilled water and DMSO with a ratio (7 : 3, respectively) while maintaining stirring at 50 °C until the formation of a clear solution. After that, the heater was turned off while retaining the stirring to allow the solution to reach room temperature. After cooling down, Irgacure 2959 (I_2959_) photoinitiator was added to the graft solution with the following concentrations (0.1%, 0.3%, and 0.5% w/v), which are coded as (GH-1, GH-2, and GH-3, respectively) at room temperature. The polymer/photoinitiator mixture solution was stirred in the absence of light to prevent premature crosslinking. Subsequently, the solution was exposed directly to a UV lamp at a 0 cm distance, at *λ* 365 nm to achieve a crosslinked G-g-GMA hydrogel. The gelation time of each solution was recorded.

0.5% w/v of I_2959_ was selected as the optimum ratio to prevent a high cytotoxic effect when increasing the concentration of the photoinitiator. Trials of increasing the redissolved graft concentration were conducted using the following concentrations (30%, 40%, and 50%). A 30% redissolved graft concentration (GH-4) was selected as the optimum concentration to proceed with, as higher concentrations did not redissolved in the solvent system.

#### 2.2.3 Preparation of Morin-loaded G-g-GMA hydrogel

Morin as a drug model was added at different concentrations (0, 1, 3, 5% (w/v)) to the optimal solution composed of redissolved graft (30%) and heated at 50 °C until fully dissolved. I_2959_ (0.5%), additional conditions were repeated at room temperature. Finally, the solution was exposed directly to a UV lamp at a 0 cm distance, at *λ* 365 nm to achieve crosslinked loaded G-g-GMA hydrogel. The gelation time of each solution was recorded. All developed hydrogels were subjected to equal intervals of time utilizing UV lamp.

### 2.3 Characterization of G-g-GMA hydrogel

#### 2.3.1 Study of swelling rate (%) of hydrogel

To evaluate their swelling rate, hydrogel samples were initially weighed (*W*_0_), before being immersed in distilled water and incubated at room temperature for 72 h. At specified time intervals (30 min, 1 h, 2 h, 3 h, 6 h, 24 h, 48 h, and 72 h), the swollen hydrogels were removed from the distilled water and weighed (*W*_1_), following the procedure outlined by.^27^ The swelling rate of the hydrogels was determined using the following equation (eqn (1)):

1 Swelling ratio (%) = ((*W*_1_ − *W*_0_)/*W*_0_) × 100%

#### 2.3.2 SEM investigation

The porosity and morphological features of the developed hydrogels were evaluated using analytical scanning electron microscopy (SEM) (Quattro S, Thermo Scientific, USA).^28,29^ A voltage of 15 kV was applied to examine both the surface and the internal structures of hydrogel matrices. Before imaging, the samples were immersed in distilled water for six hours to allow swelling, a step designed to improve imaging quality and provide more precise visualization of the hydrogel's porosity. Following this, the hydrogel membranes were dehydrated using a lyophilizer (Christ freeze dryer, alpha 1–4, Germany).

#### 2.3.3 FTIR analysis

FT-IR analysis was accomplished using FTIR spectrometer (Bruker Vertex 70, Germany) to examine the chemical composition of oven-dried hydrogel samples and their components.^30,31^ The spectral data were collected within *ν* 4000–400 cm^−1^ to identify the characteristic peaks corresponding to the hydrogel components.

### 2.4 *In vivo* investigation

#### 2.4.1 Animal grouping and housing

The *in vivo* experiment was approved by the Badr University in Cairo, Institutional Ethical Committee No. (BUC-IACUC-250218-128). 12 male Wistar rats, weigh 230–250 g, were separated into cages. Rats were kept under controlled temperature (21  ± 5 °C), free access to water and food, and maintained at a 12-h light/dark cycle.

#### 2.4.2 Experimental

Ketamine (80 mg kg^−1^) was used to anesthetize rats, and their hair was then removed.^32^ The dorsal region was carefully shaved to expose the skin surface, and a metal rod with a diameter of 1 cm^2^ was heated.^33^ This rod was then applied to the shaved dorsal skin for 5 seconds to create a 2nd degree burn wound. Surgical gauges were used for all rats, and they were kept in their cages to avoid infection. Group 1 was kept without treatment and served as a negative control. Group II was treated with hydrogel as the positive control, every three days, and Group III was treated with 5% Morin-loaded hydrogel (GH-7), every three days as well. After 3, 7, 14, 17, and 21 days, the wound dressings were removed, and photographs of the wounds were taken for documentation. The diameters of the scars were measured with a caliper, and the wound healing (%) was assessed according to eqn (2):2
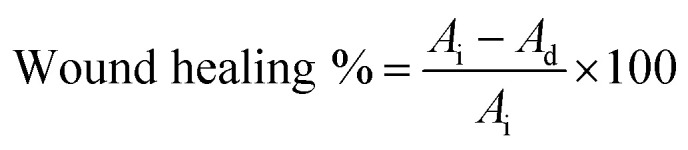
where *A*_d_ and *A*_i_ represent the burn areas on the specified day and day zero, respectively. The rats were sacrificed on day 21. To preserve the skin samples for later histological analysis, they were immediately placed in 10% formalin.

#### 2.4.3 Histopathological assessment

Biopsies of burn sites and normal skin from all groups were fixed in 10% phosphate-buffered formalin, and 3–5 µm sections were prepared. Sections were used for hematoxylin and Eosin staining (H&E).^34^ The features of normal skin included an intact covering epithelium, preserved skin adnexa in the form of pilosebaceous units within the dermis, and organized dermal collagen bundles oriented parallel to the covering epithelium. In contrast, skin affected by burns exhibited ulceration, either complete or focal, of the covering epithelium, loss of skin adnexa with absent pilosebaceous units, and replacement of collagen fibers by granulation tissue. Evidence of healing in burn sites was characterized by partial or complete epithelialization, the replacement of granulation tissue by collagen fibers initially disorganized but later becoming organized with a parallel orientation to the surface epithelium and the development of skin adnexa, including hair follicles and sebaceous glands.

### 2.5 Cell cultures and stimulation

THP-1, a human monocytic leukemia line, was acquired from American Type Culture Collection (ATCC) and cultured in RPMI-1640 medium, supplemented with 10% fetal bovine serum, 2 mM glutamine. 1 mM sodium pyruvate, 10 mM HEPES, and antibiotics (50 µg mL^−1^ streptomycin and 50 U per mL penicillin).

#### 2.5.1 Macrophage THP-1 differentiation and stimulation

A macrophage polarization model was set up, starting from THP-1 monocytes differentiated into macrophages using PMA (Phorbol 12-myristate 13-acetate). Once differentiated, the M0-macrophages were then stimulated using interferon-gamma (IFN-γ) and lipopolysaccharides (LPS) for classical macrophage activation (M1).^35^ Following stimulation, the M1-macrophages were incubated with various hydrogel composites to study their impact on the macrophage functions. The levels of nitric oxide (NO), cytokines IL-1β and IL-6 were estimated as described below. The transcription levels of inducible nitric oxide synthase (iNOS), cyclooxygenase-2 (COX-2), and Stat-3 genes were evaluated using RT-PCR.

#### 2.5.2 Measurement of nitric oxide (NO) production

According to the manufacturer's instructions, THP-1 cell lysate's nitric oxide level was assessed after cellular stimulation with IFN-γ (10 ng mL^−1^), LPS (10 ng mL^−1^), alone or in combination, using a commercial NO-kit (Cat. No. KGE001, R&D Systems Inc). THP-1 macrophages were plated in a 24-well plate at a density of 6 × 10^4^ cells per well. Following a 24-hour incubation, cells were pre-treated with 0.1 µg mL^−1^ LPS. The plate was then incubated for 24 hours at 37 °C with 5% CO_2_. After incubation, the supernatant from each well was transferred to a 96-well plate and mixed with a 1 : 1 combination of Griess reagents A and B. Nitric oxide production was quantified by recording absorbance at 550 nm after 15-minute incubation.^36–38^

#### 2.5.3 Estimation of cytokines

THP-1 cells were stimulated by IFN-γ (10 ng mL^−1^), LPS (10 ng mL^−1^), alone or in combination with different hydrogel. Subsequently, IL-1β and Il-6 protein level was assessed using human IL-6 and IL-1β ELISA kit and according to the manufacturer's protocol. Briefly, 96-well microplate was precoated with capture antibody (100 µL per well) during overnight. After washing 3 times, the plates were blocked by 300 µL of reagent diluent and incubated at room temperature for 1 h. Diluted standards, controls, and samples were added to duplicate wells (100 µL per well), and plates were left at room temperature for 2 h. Following washing 3 times, Streptavidin-HRP was added (100 µL per well) and incubated for 20 minutes at room temperature, protected from light. Next, 100 µL of substrate solution was added per well for another 20-minute incubation. The reaction was stopped by adding 50 µL of stop solution to each well, and the plate was gently tapped to ensure thorough mixing, absorbance was immediately measured at 450 nm.^39,40^

#### 2.5.4 Quantitative real-time PCR analysis

The quantitative real-time PCR protocol was adapted from established methods. THP-1 cells were plated in 6-well plates and incubated for 24 hours under standard conditions (37 °C, 5% CO_2_). Following this, the cells were exposed to a non-cytotoxic concentration of the hydrogel formulation alongside a co-stimulus of IFN-γ (10 ng mL^−1^) and LPS (10 ng mL^−1^). Untreated cells were included as a blank control. A negative control (LPS group) consisted of cells stimulated with IFN-γ/LPS in the presence of the vehicle DMSO at a final concentration of 0.1% (v/v), without any hydrogel. After a 24-hour incubation period, total RNA was isolated using Trizol Reagent, strictly following the manufacturer's instructions.^41^ It was reverse transcribed into complementary DNA. The real-time RT-PCR was performed in a 20 µL volume using a SYBR Green PCR Core Reagent Kit.^42^ The relative expression of each gene was calculated by the Livak method or 2^−ΔΔCT^ method, normalized to GAPDH. All primers utilized in this experiment are provided in Table 1.

**Table 1:** Primer séquences for quantitative RT-PCR

Gene name	Sense primer	Antisense primer
iNOS	CAGGAGGAGAGAGATCCGATTTA	GCATTAGCATGGAAGCAAAGA
COX-2	GAAGATTCCCT CCGGTGTTT	CCCTTCTCACTGGCTTATGTAG
STAT-3	CTTTGAGACCGAGGTGTATCACC	GGTCAGCATGTTGTACCACAGG
GAPDH	GGCCTTCCGTGTTCCTAC	TGTCATCATATCTGGCAGGTT

### 2.6. Statistical analysis

Data was collected from a minimum of three independent experiments and presented as means ± standard deviations. Statistical analysis was performed using a one-way analysis of variance (ANOVA) with a significance level of *p* ≤ 0.05. The analysis was conducted using SPSS software (IBM® SPSS®, version 27). Table 2 represents the coding system used in this study.

**Table 2:** Formulation codes and concentrations of evaluated hydrogels

Sample codes	Content
GH-1	20% graft + 0.1% I_2959_
GH-2	20% graft + 0.3% I_2959_
GH-3	20% graft + 0.5% I_2959_
GH-4	30% graft + 0.5% I_2959_ + 0% Morin (control)
GH-5	30% graft + 0.5% I_2959_ + 1% Morin
GH-6	30% graft + 0.5% I_2959_ + 3% Morin
GH-7	30% graft + 0.5% I_2959_ + 5% Morin

## 3. Results and discussion

### 3.1 Determination of gelation time of hydrogels

**Fig. 1:**
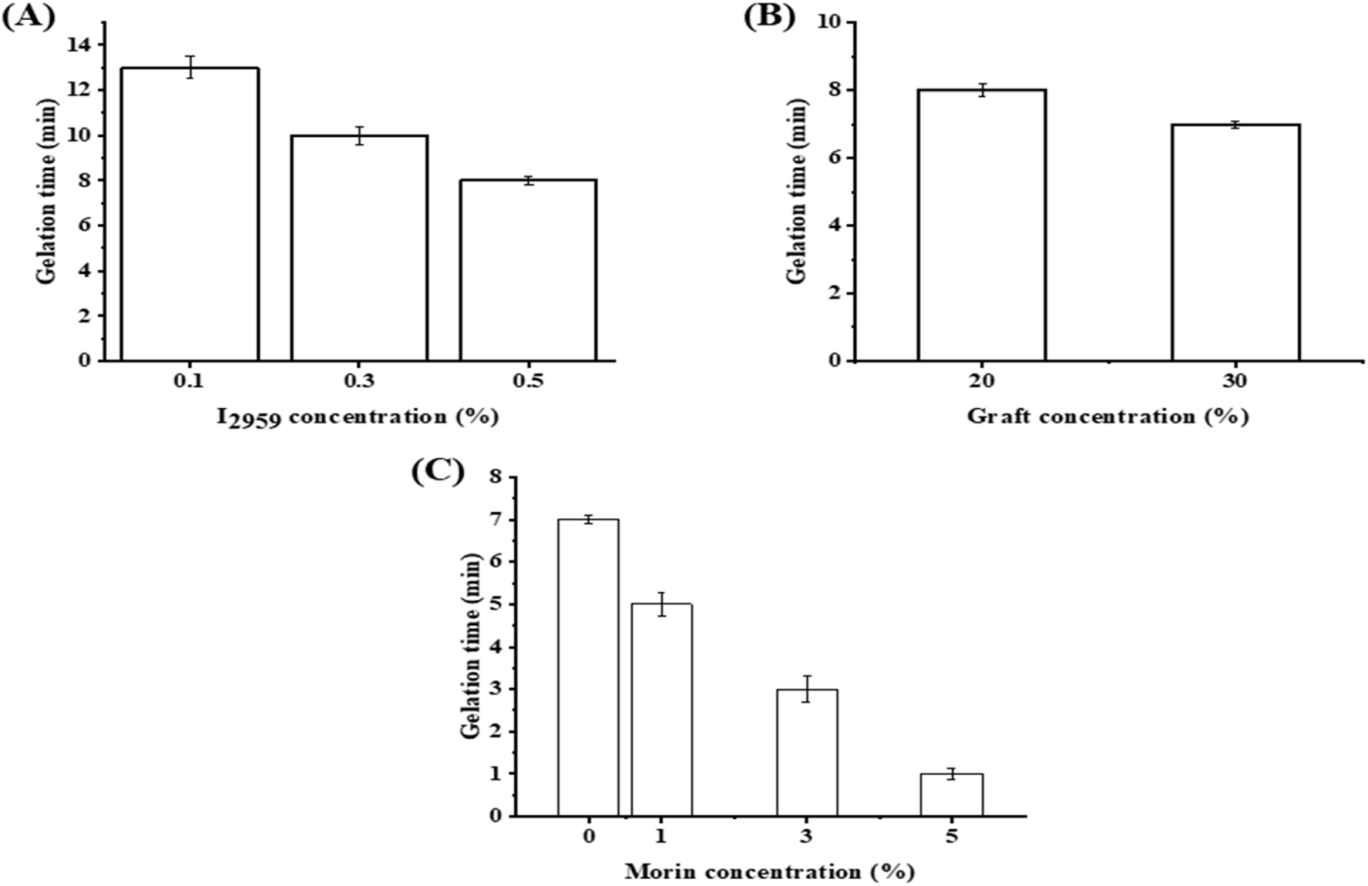
Gelation time of G-g-GMA hydrogels of; (A) fixed 20% graft and different I_2959_ conc. (from GH-1 to GH-3, respectively). (B) 20% redissolved graft *vs.* 30% graft with fixed 0.5% I_2959_ (GH-3, and GH-4, respectively). (C) Loading Morin to G-g-GMA hydrogel matrix at various concentrations (from GH-4 to GH-7, respectively).

The determination of the gelation time of the hydrogel is crucial for the hydrogel preparation. Fig. 1 illustrates the impact of several parameters on the hydrogel gelation time. Fig. 1A reveals the impact of various concentrations of I_2959_ (w/v) on the hydrogel gelation time. As shown, the increase in photoinitiator concentration led to a decrease in the gelation time of the hydrogel, reaching approximately eight minutes at a 0.5% (w/v) concentration. These results are additionally confirmed by previous results,^43^ which demonstrate an inverse proportion relation between the increase in the photoinitiator concentration and the decrease in gelation time. Further increases in the concentration of the photoinitiator were not carried out to avoid the associated increase in the cytotoxic effect of photoinitiator. The concentration of 0.5% of I_2959_ demonstrated the optimal gelation time compared to lower concentrations of the photoinitiator. The methacrylate groups of GMA undergo radical polymerization upon exposure to UV light, with the radicals generated by the photoinitiator. Fig. 1B demonstrates the effect of increasing the redissolved graft concentration on the gelation time of the hydrogel. Increasing the graft concentration to 30% (w/v) results in a reduced gelation time, attributed to the higher number of crosslinked bonds formed during hydrogel development.^44^ The sample, with enhanced gelation time, was selected to serve as the control in further experiments. Fig. 1C reveals the effect of addition of various concentrations (w/v) of Morin on the hydrogel gelation time. As shown in the results, the gelation time was enhanced and decreased after adding various concentrations of the Morin to the hydrogel matrix. The 5% Morin addition yielded the quickest gelation time (∼one min) until full gelation was achieved. The enhanced gelation time was attributed to the H-bonds formed between the drug and the grafted hydrogel.^45^ This highlights its potential to enhance the hydrogel's properties effectively.

### 3.2 Determination of swelling rate (%) of hydrogels

**Fig. 2:**
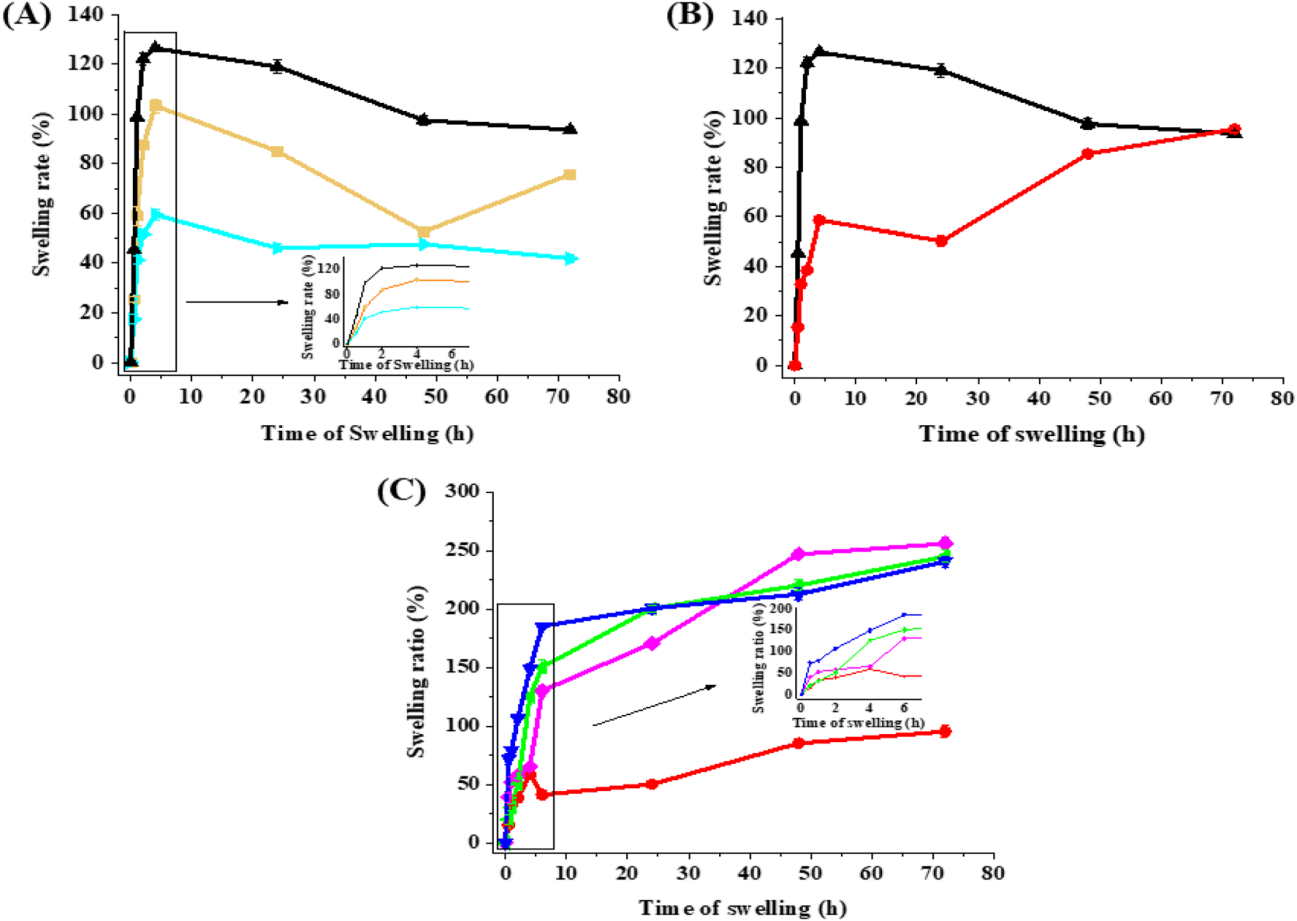
Swelling ratio of G-g-GMA hydrogels. (A) Swelling ratio of GH-1 (■) (Brown), GH-2 (▶) (Cyan), and GH-3 (▲) (Black). (B) Swelling ratio of GH-3 (▲) (Black), and GH-4 (●) (Red). (C) GH-4 (●) (Red) and after including Morin at concentrations of 1% (GH-5) (♦) (Purple), 3% (GH-6) (◄) (Green), and 5% (GH-7) (▼) (Blue).

Hydrogels with a high swelling capacity are widely used in various applications, including wound healing, drug delivery, and tissue regeneration.^46^ Swelling aids in the absorption of blood and wound exudates, facilitating the transfer of nutrients and metabolites and cell infiltration.^47^ Hydrogels' swelling is an essential feature to determine their structural integrity and their stability in aqueous solution. The developed scaffolds demonstrated hydrophilic nature due to water uptake.^48^Fig. 2A illustrates the effect of various concentrations of I_2959_ (w/v) on the swelling rate of hydrogel. As shown in the figure, 0.5% (w/v) I_2959_ demonstrated the highest swelling percentage (∼126%) in comparison to the lower concentrations of the photoinitiator, which is needed for the hydrogel to transport nutrients and drug release. Fig. 2B illustrates the effect of redissolving 20% of the graft *versus* 30% graft with a fixed concentration of 0.5% I_2959_ on the swelling percentage of the hydrogel. The swelling percentage decreased when increasing the graft concentration which is attributed to the increased mechanical bonds between the hydrogel matrix which enhance the stability of hydrogel against degradation due to excessive swelling in lower redissolved graft concentrations. Fig. 2C demonstrates the impact of the addition of Morin on the hydrogel swelling ratio. Morin improved the swelling ratio of the hydrogel which reported (∼240%) in comparison to the free hydrogel which is attributed to the hydrophilicity of Morin.

### 3.3 SEM investigation of hydrogels

**Fig. 3:**
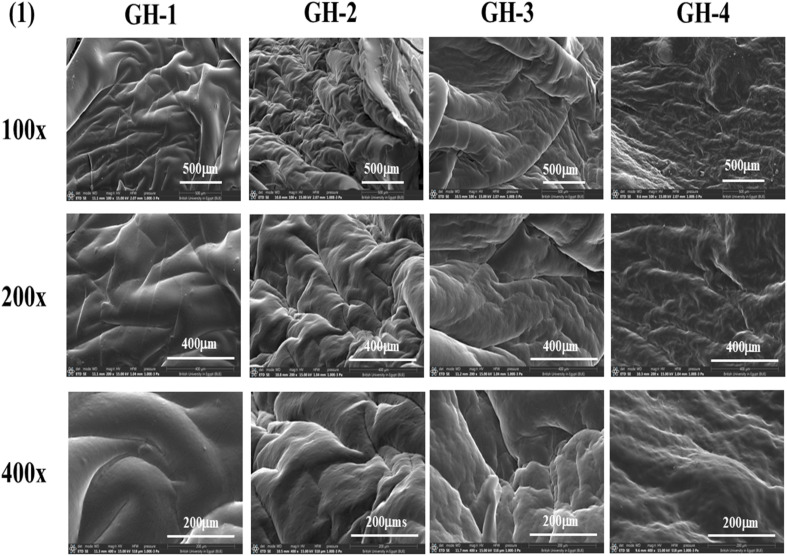
SEM images of four different gel formulations, GH-1, GH-2, GH-3, and GH-4 (control formulation), at various magnifications. Each sample was imaged with three different magnifications, 100×, 200×, and 400×, at 10 kV. The corresponding images' scale bars are included on the images.

SEM was utilized to examine the surface morphology and porosity of hydrogel membranes{Kamoun, 2025 #66}. Fig. 3 demonstrates successful crosslinking between gelatin and GMA when adding different concentrations of photoinitiator to 20% redissolved graft, which resulted in the formation of a convolution texture in the hydrogel matrix characterized by the presence of thick walls. As shown, 0.5% I_2959_ demonstrates enhanced convolutions to hydrogel morphology. This concentration was fixed for the following procedure, as increasing the photoinitiator concentration increases its cytotoxic characteristics.^49^ In Fig. 3, GH-4 represents the addition of 0.5% of the I_2959_ to 30% of the redissolved graft to allow for the formation of the hydrogel. The increased redissolved graft concentration demonstrated enhanced convolutions in the matrix of the hydrogel; therefore, this sample will serve as the control sample for the rest of the experiments.

**Fig. 4:**
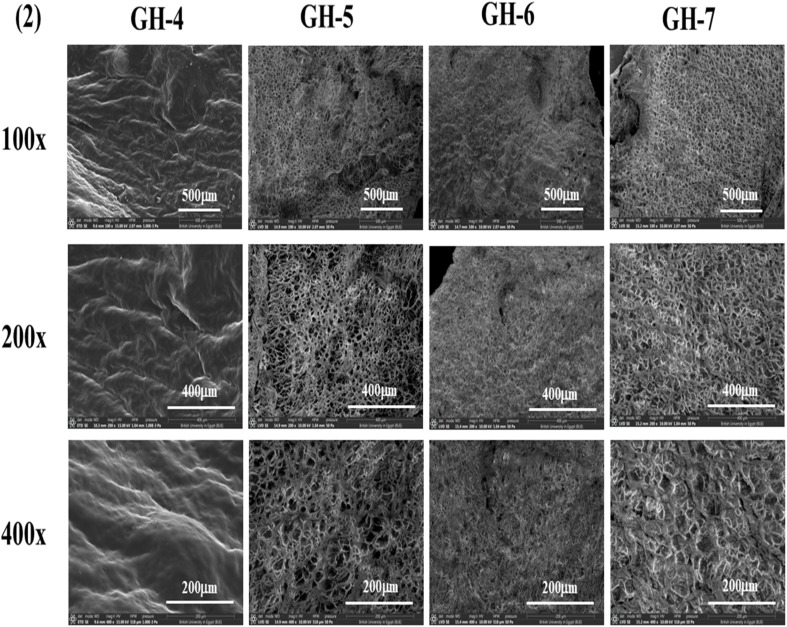
SEM images of four different gel formulations, GH4 (control formulation), GH-5, GH-6, and GH-7, at various magnifications. Each sample was imaged with three different magnifications, 100×, 200×, and 400×, at 10 kV. The corresponding images' scale bars are included on the images.

In Fig. 4, GH-4 demonstrated successful crosslinking between the gelatin and GMA, when adding different concentrations of the Morin to the control sample. The addition of the Morin significantly enhanced the matrix morphology which was shown as the formation of a porous hydrogel structure (GH5, GH6, and GH7). As represented in the Fig. 4, the increase in the concentration of the drug resulted in enhanced porosity and demonstrated enhanced homogeneity. The enhanced porosity allows for increased cellular migration and proliferation, and penetration of the growth factor to the site of injury, thus enhancing the therapeutic benefits of the hydrogel.^50^

### 3.4 FTIR analysis of hydrogels

**Fig. 5:**
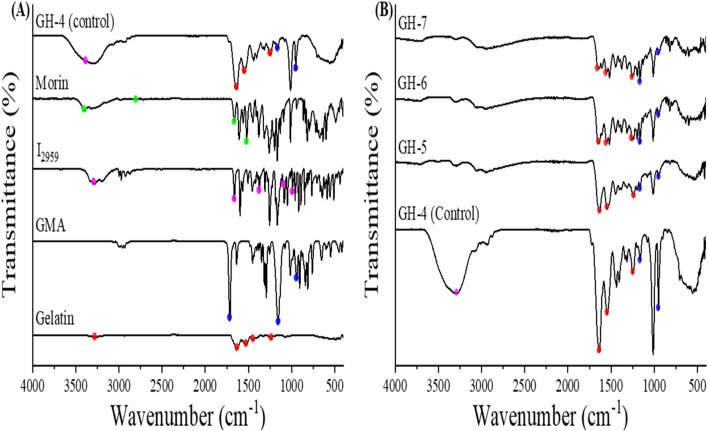
FTIR spectra of G-g-GMA hydrogel and individual components of hydrogels (A) Gelatin (Red), GMA (Blue), I_2959_ photoinitiator (Purple), Morin (Green) and GH-4 (Control). (B) FTIR spectra of GH-4, GH-5, GH-6 and GH-7.

The functional groups of hydrogels' components were identified using FTIR to determine chemical interactions (Fig. 5). FTIR spectrum of gelatin showed peaks at *ν* 1630, 1528, and 1446 cm^−1^ corresponding to –C=O stretching of amide I, –C–H stretching of amide II, and methyl group, respectively, and broad peak from *ν* 3394 to 3165 cm^−1^, owing to –N–H stretching of amide.^51^ For GMA spectrum it demonstrated C=O, owing to methacryloyl groups at *ν* 1715 cm^−1^, out-of-plan bending of R_2_C=CH_2_ group at *ν* 942 cm^−1^ and stretching of the C–O (ester) group at *ν* 1160 cm^−1^. Further, C=C stretching of the methacrylate group, and possibly C=O stretching from the carbonyl groups. The spectrum does shift; this could indicate the presence of polymerized methacrylate groups in the hydrogel. The I_2959_ demonstrated distinctive peaks at the bands *ν* 1371 cm^−1^ (C–H wagging vibration, CH_2_) and *ν* 1011 cm^−1^ (C–H wagging vibration, CH_3_), while the band with the peak at *ν* 1665 cm^−1^ (C=O stretching vibration). Additionally, the band that has a peak at *ν* 988 cm^−1^ (phenyl ring, C–H out-of-plane vibration).^52^ The following Morin distinctive peaks were observed at *ν* 1528–1518 cm^−1^ (stretching C–C vibrations of aromatic ring), *ν* 1650 cm^−1^ (ketone CO stretching), a broad peak from ∼ *ν* 3200 to 3400 cm^−1^ for OH group, and 2915 cm^−1^ (C–H stretching).^53^

Fig. 5B illustrates the spectra for the unloaded optimized hydrogel (GH4) alongside the loaded hydrogels containing varying concentrations of Morin (GH5, GH6, and GH7). It was observed that only crosslinked Gelatin-G-GMA hydrogel (GH4) exhibits a distinct band containing a carbonyl ester group at *ν* 1719.5 cm^−1^.^19^ Additionally, it was observed in the loaded hydrogels that the Morin peaks are not clearly visible, indicating incorporation of Morin inside the hydrogel matrix. The final hydrogel confirmed the successful incorporation of its components into the matrix by retaining the characteristic peaks of each component.

### 3.5. Wound healing rate analysis

**Fig. 6:**
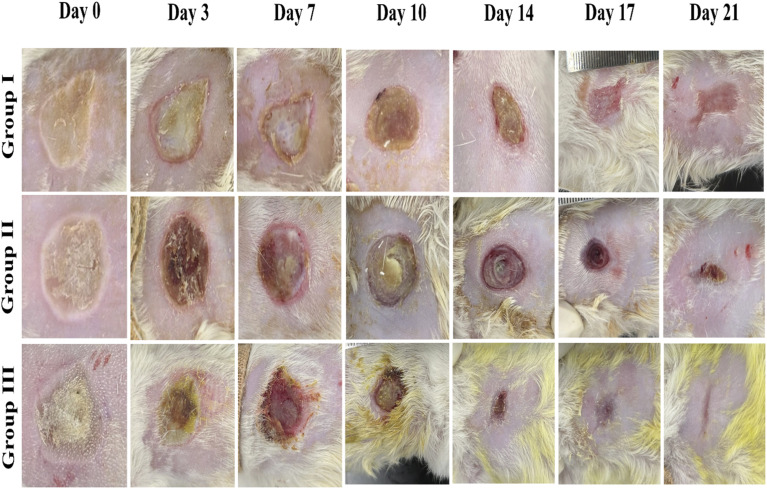
*In vivo* burn wound healing was observed primarily by creating burn wound injury and treating it. Negative control: no treatment (group I), positive control: unloaded hydrogel (group II), and Morin-loaded hydrogel (group III) on different days (3, 7, 10, 14, 17, and 21 days).

**Fig. 7:**
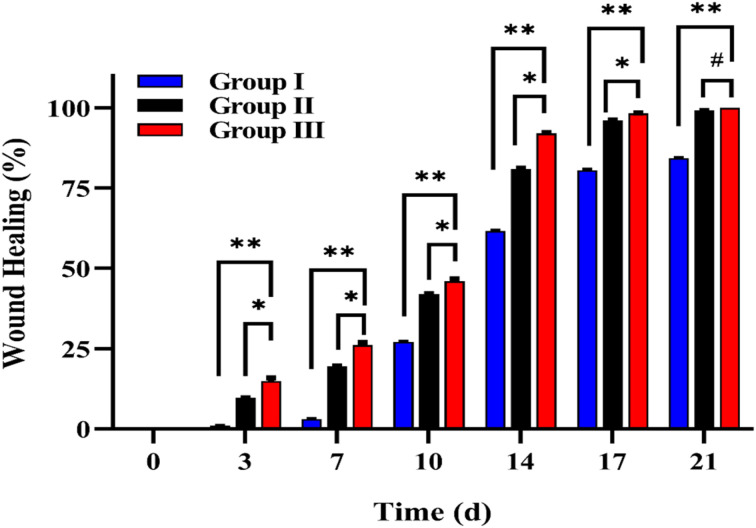
Wound healing percentages (%) on days 3, 7, 10, 14, 17, and 21 in group I (negative control), group II (positive control: treated with unloaded hydrogel), and group III (treated with Morin-loaded hydrogel). **, *, # refer to statistical differences compared to group I at *p* < 0.0001, group II at *p* < 0.0001, and group II at *p* < 0.0026, respectively.

In this study, a burn model was applied to assess the healing capacity of the Morin-loaded hydrogels. The burn progression was systematically recorded at specific intervals (0, 3, 7, 10, 14, 17, and 21 d), as depicted in Fig. 6. The treated groups (groups II and III) displayed a significantly higher percentage of wound healing than the control group (group I). As shown in Fig. 7, group III showed the highest % wound healing among all tested groups. The positions treated with the Morin-loaded hydrogel on rats showed continuous burn edge contraction during treatment. By the end of the treatment, Group III reached the maximum contraction of the burn diameter. This indicates that the Morin-loaded hydrogel formula showed the best healing properties compared to the hydrogel and the untreated groups. By day 17, Group III demonstrated an impressive wound healing percentage of *ca.* 98% outperforming the other groups. By day 21, Group III achieved nearly complete wound closure (*ca.* 99.9%), whereas the control group reached a closure rate of 84%.

### 3.6. Histopathology study

**Fig. 8:**
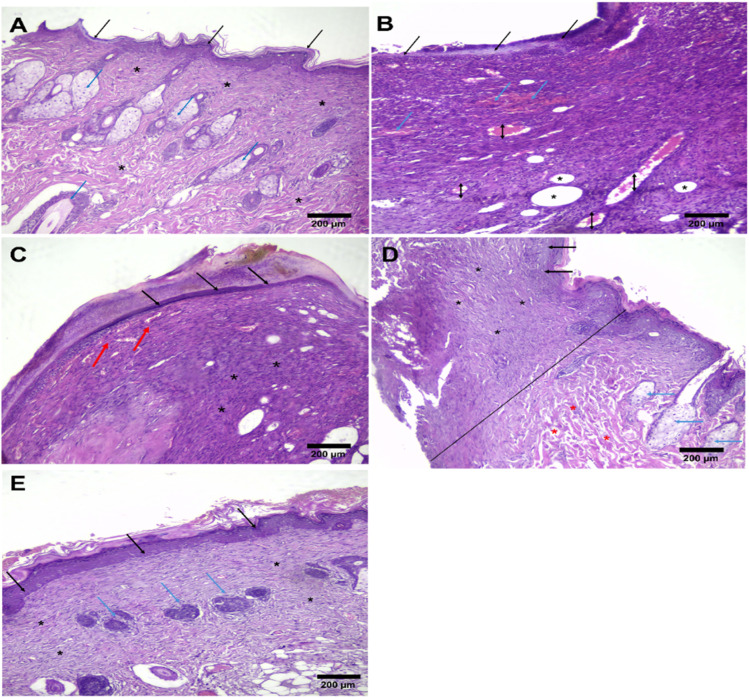
Histopathological examination (H&E) for (A) normal skin (healthy control), (B) second-degree burn of the skin at day 0, (C) group I (negative control), (D) group II (positive control), (E) group III (the burnt area at the end of the experiment). Where normal intact keratinized squamous epithelium (black arrows). The dermis shows preserved skin adnexa in the form of sebaceous glands and hair follicles (blue arrows). The underlying dermis is replaced with granulation tissue in the upper half (red arrows). Dermal collagen fibres show normal histologic orientation (*).

As shown in Fig. 8, Morin-loaded hydrogel (group III) promotes healing effects of second-degree burns, which can be evidenced through complete tissue epithelialization and the development of skin adnexa in the form of primitive hair follicles and sebaceous glands. Additionally, there is no residual granulation tissue, which is entirely replaced by collagen fibers with organized parallel orientation to the covering epithelium. The healing impact of Morin in wound healing in group III seems to be more efficient than that of unloaded hydrogel in the positive control group (group II), where skin adnexa are not developed yet, and collagen fibers are still disorganized.

Considering the burn context and its management, Morin, a naturally occurring flavonoid, can play a significant role in achieving effective wound healing by modulating the activity of fibroblasts and the regeneration of epithelial cells. This could be achieved by understanding that fibroblasts play a significant role in wound healing by synthesizing the extracellular matrix, including collagen. After encountering a burn injury, Morin can stimulate fibroblasts proliferation, which enhances the required formation of granulation tissue and closing the wound. This could lead to the stimulation of the formation of collagen and facilitate tissue integrity in the process of healing. Even though activation of fibroblasts is required for wound healing, their over-activation will result in the production of too much collagen, leading to pathological fibrosis, which is presented as hypertrophic scarring, a common complication in burn patients. Morin has been demonstrated to exhibit anti-fibrotic activity in research, whereby it is able to modulate collagen production and inhibit its overproduction, thereby decreasing the likelihood of abnormal scarring and enhancing cosmetic and functional recovery in burn injuries.^54^ Moreover, Morin's impact on epithelial architecture plays also a significant role in the treatment of burns. Epidermal and dermal epithelial regeneration of the skin is required for the restoration of the skin's protective function and for the minimization of the risk of infection, two of the serious issues associated with burns' healing.^7,55^ The antiinflammatory and antioxidant activities of Morin protect epithelial cells from oxidative stress and inflammation, both induced by burn injury. In addition to blocking oxidative stress, Morin also could promote epithelial cell growth and migration necessary for re-epithelialization, the process of wound healing that repopulates the skin surface following injury. The promotion of epithelial regeneration by Morin can accelerate wound closure, minimize infection, and improve the healing process. In total, Morin possesses great potential in burn healing through fibroblastic activity induction that is critical in wound healing without causing excessive scarring. Its antioxidants, anti-inflammatory, and anti-fibrotic properties account for improved epithelial regeneration, quicker wound closure, and reduced fibrosis.^56^

### 3.7. Effect of different Morin hydrogel composites on inflammatory mediators in M1 macrophages

#### 3.7.1. Suppression of M1 macrophage nitric oxide production by Morin-loaded hydrogels

**Fig. 9:**
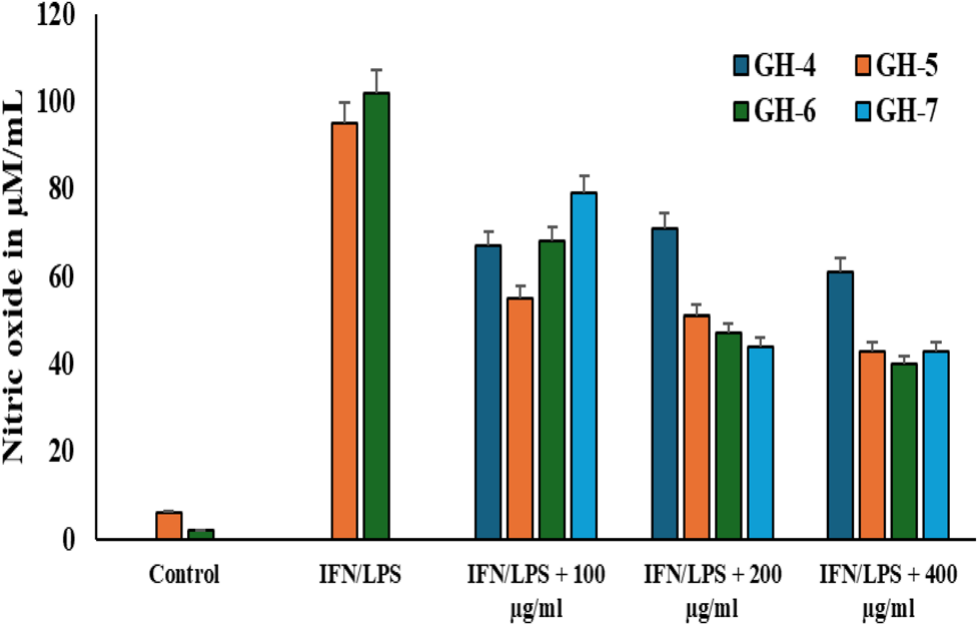
Effect of different hydrogel composites on nitric oxide production in M1 macrophages.

The THP-1-derived M1 macrophage model is a standardized and well-characterized *in vitro* model to study inflammation and to screen new therapeutic compounds.^57^ It can be used to investigate the efficacy of anti-inflammatory agents by measuring their ability to suppress the hallmark M1 outputs (NO, IL-6, IL-1β, TNF-α…. *etc*). The macrophages were stimulated and polarized with IFN-γ/LPS to induce a classical pro-inflammatory (M1) state before being treated with increasing concentrations (100, 200, and 400 µg mL^−1^) of extracts from hydrogel formulations GH-4, GH-5, GH-6, and GH-7. Nitric Oxide, key gaseous inflammatory mediator, was measured in the cell culture supernatant as shown in Fig. 9. Moreover, it was observed that stimulation with IFN-γ/LPS induced the M1 phenotype, resulting in a significant increase in the production of Nitric Oxide compared to the untreated Control group in the cell culture supernatant. This confirms the successful establishment of the M1 inflammatory model. Higher concentrations of the hydrogel extracts (400 µg mL^−1^) led to significant reduction of NO levels compared to lower concentrations (100 and 200 µg mL^−1^). GH-4 (Control Hydrogel: 30% graft + 0.5% I2959) caused a moderate reduction of NO, suggesting the base hydrogel material itself possesses some inherent anti-inflammatory properties. While GH-5, GH-6, GH-7 with Morin at 1%, 3%, and 5% respectively, showed a significantly stronger suppressive effect than GH-4. GH-7 with 5% Morin exhibited the most potent effect, with its 400 µg mL^−1^ extract likely reducing NO levels closest to the baseline control. This indicates that the anti-inflammatory potency is enhanced by higher loading of Morin within the hydrogel.

#### 3.7.2. Suppression of M1 macrophage pro-inflammatory cytokines and key inflammatory genes by Morin-loaded hydrogels

**Fig. 10:**
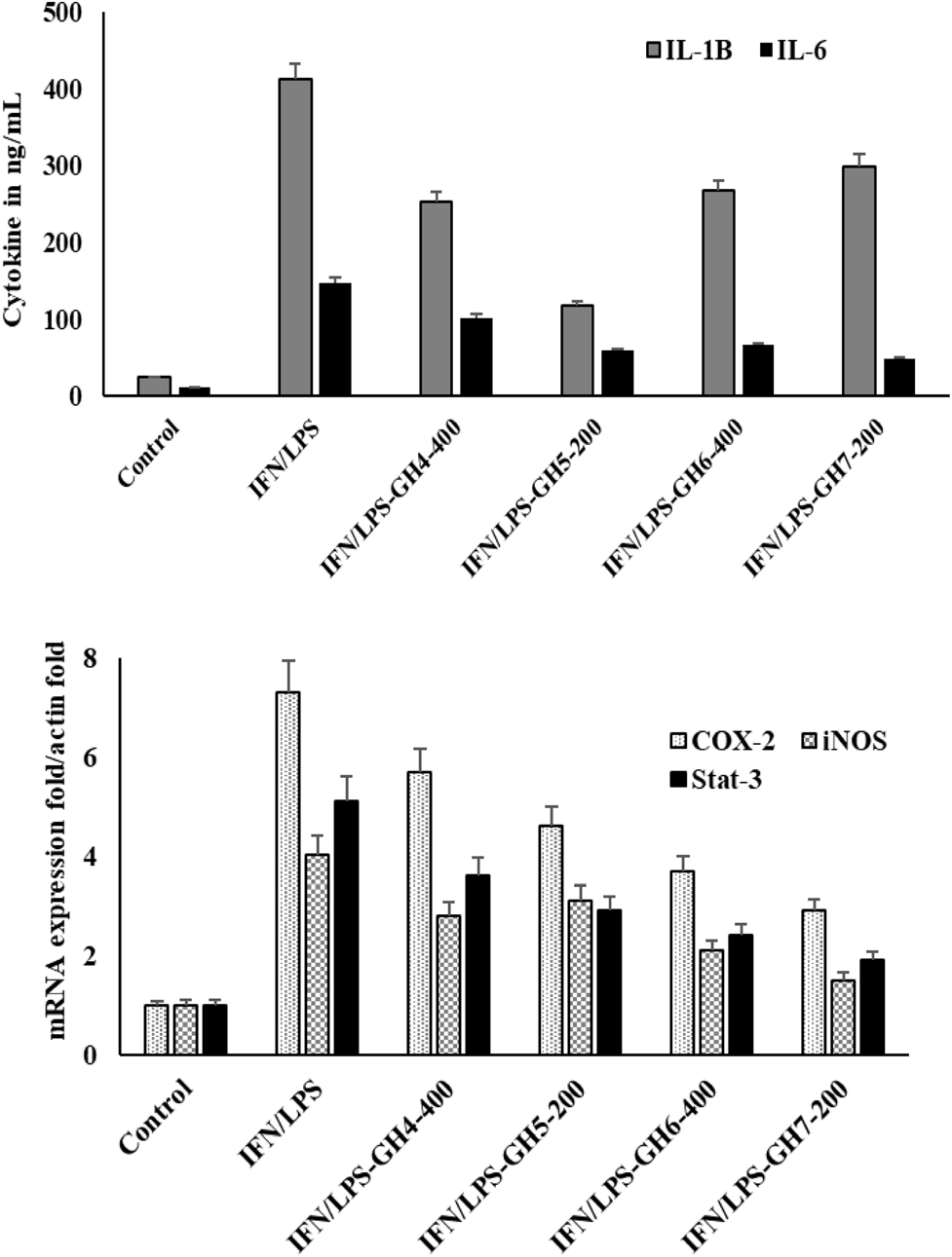
Effect of different hydrogel composites on inflammatory mediators in M1 macrophages.

To elucidate the mechanism behind the reduced nitric oxide production, the anti-inflammatory impact of the hydrogel composites was further validated by analyzing cytokine secretion (IL-1β and IL-6) and key inflammatory genes expression (COX-2, iNOS and STAT-3) in M1 macrophages (Fig. 10). Macrophages were first polarized to the M1 phenotype with IFN-γ/LPS stimulation and then treated with specific concentrations of extracts from hydrogel formulations GH-4, GH-5, GH-6, and GH-7. Upon incubation with IFN-γ/LPS, a dramatic increase in both cytokine secretion (IL-1β, IL-6) and gene expression (COX-2, iNOS, STAT-3) compared to the untreated Control, confirming a robust M1 inflammatory response. The Morin-containing hydrogels (GH-5, GH-6, GH-7) show a stronger suppressive effect compared to the control hydrogel GH-4. The effect appears concentration-dependent, with higher extract doses (400 µg mL^−1^ of GH-7) showing greater suppression than lower doses (200 µg mL^−1^ of GH-5 and GH-7).

It is noticed that hydrogel operates not only by reducing the secretion of potent pro-inflammatory cytokines (IL-1β, IL-6) but also by suppressing the genetic programming that drives the M1 inflammatory response. The downregulation of iNOS, COX-2, and STAT-3 mRNA transcription provides a direct mechanistic explanation for the reduced NO production.

The results confirm that efficacy is influenced by both the presence of Morin and the concentration of the hydrogel extract, with GH-7 at 400 µg mL^−1^ likely showing one of the strongest effects. This confirms that these hydrogel composites are potent modulators of macrophage-mediated inflammation, acting at the fundamental level of gene expression.

## 4. Conclusions

In the current study, we developed Morin loaded gelatin-g-GMA based photopolymerized hydrogel as a promising scaffold for promoting burn wound healing. Composite hydrogel was shown to possess superior physicochemical characteristics such as mechanical strength, swelling behavior, and biodegradability that render it a suitable scaffold material for tissue engineering. Morin loading greatly enhanced the proliferation of fibroblasts and collagen synthesis necessary for tissue repair and regeneration, as evidenced *in vivo*. In addition, Morin's anti-inflammatory and anti-fibrotic effects reduced excessive scarring and promoted a more favorable healing environment. The hydrogel was also promising in facilitating epithelial regeneration, which is critical to restoration of skin protective barrier function following burn injury. Morin-loaded gelatin-GMA hydrogel is a new way to induce tissue regeneration without contributing to risk of hypertrophic scarring and contractures, both of which are common burn healing complications. Following completion of treatment, group III experienced maximum contraction of the burn diameter, and this reaffirmed that Morin-loaded hydrogel formulation exhibited higher healing efficiency compared to hydrogel alone and control groups. The developed Morin-loaded hydrogel composites, particularly GH-7, emerge as highly effective anti-inflammatory agents by potently suppressing M1 macrophage phenotype. Their efficacy is demonstrated through the significant, dose-dependent inhibition of critical inflammatory mediators (NO, IL-1β, IL-6) and fundamental downregulation of associated genes (iNOS, COX-2, STAT-3). These findings confirm that encapsulating Morin within a hydrogel matrix enhances its bioactivity and presents a promising therapeutic strategy for modulating macrophage-driven inflammatory pathologies. In general, developed hydrogel offers a platform to fabricate the next generation of biomaterials that will potentially re-engineer burn care and tissue engineering, leading to more efficient and individualized therapeutic solutions to burn treatment and regenerative medicine.

## Ethical statement

The *in vivo* experiment was approved by the “Badr University in Cairo, Institutional Ethical Committee No. (BUC-IACUC-250218-128).

## Author contributions

Tasneem Abed: methodology, formal analysis, and data analysis. Samar A. Salim: methodology, formal analysis, resources, reviewed the final draft. Yasser A. Elnakady, Jong Yeog Son, Ahmed I Ali: resources, software, data analysis and reviewed the final draft. Marwa Mosaad Shakweer, Esraa B. Abdelazim: *In vivo* and histopathology work; Elbadawy A. Kamoun, Amr Negm and Mahmoud Elsabahy: project management, supervision, wrote the original draft and reviewed the final draft. All authors approved the current and final version of the manuscript for submission.

## Conflicts of interest

There are no competing interests.

## Funding

This work was supported by the “Deanship of Scientific Research, Vice Presidency for Graduate Studies and Scientific Research, King Faisal University, Saudi Arabia [Project No. KFU260263]”.

## Data Availability

The data sets used and/or analyzed during the current study are available from the corresponding authors on reasonable request.
